# Protein engineering reveals that gasdermin A preferentially targets mitochondrial membranes over the plasma membrane during pyroptosis

**DOI:** 10.1016/j.jbc.2023.102908

**Published:** 2023-01-13

**Authors:** Hannah C. Kondolf, Dana A. D'Orlando, George R. Dubyak, Derek W. Abbott

**Affiliations:** 1 Department of Pathology, Case Western Reserve University, Cleveland, Ohio, USA; 2 Department of Physiology and Biophysics, Case Western Reserve University, Cleveland, Ohio, USA

**Keywords:** GSDMD, GSDMA, pyroptosis, cell death, mitochondria, plasma membrane, protein engineering, protein–lipid interactions, BSA, bovine serum albumin, GSDMA, gasdermin A, GSDMD, gasdermin D, HEK293T, human embryonic kidney 293T cell line, LCIS, Live Cell Imaging Solution, LDH, lactate dehydrogenase, mtDNA, mitochondrial DNA, NLRP3, NOD-, LRR-, and pyrin domain–containing protein 3, OMM, outer mitochondrial membrane, PARP, poly(ADP-ribose) polymerase, PI, propidium iodide, PMA, phorbol 12-myrisate 12-acetate, SSc, systemic sclerosis, TBST, Tris-buffered saline with Tween-20

## Abstract

When activated, gasdermin family members are thought to be pore-forming proteins that cause lytic cell death. Despite this, numerous studies have suggested that the threshold for lytic cell death is dependent on which gasdermin family member is activated. Determination of the propensity of various gasdermin family members to cause pyroptosis has been handicapped by the fact that for many of them, the mechanisms and timing of their activation are uncertain. In this article, we exploit the recently discovered exosite-mediated recognition of gasdermin D (GSDMD) by the inflammatory caspases to develop a system that activates gasdermin family members in an efficient and equivalent manner. We leverage this system to show that upon activation, GSDMD and gasdermin A (GSDMA) exhibit differential subcellular localization, differential plasma membrane permeabilization, and differential lytic cell death. While GSDMD localizes rapidly to both the plasma membrane and organelle membranes, GSDMA preferentially localizes to the mitochondria with delayed and diminished accumulation at the plasma membrane. As a consequence of this differential kinetics of subcellular localization, N-terminal GSDMA results in early mitochondrial dysfunction relative to plasma membrane permeabilization. This study thus challenges the assumption that gasdermin family members effect cell death through identical mechanisms and establishes that their activation in their respective tissues of expression likely results in different immunological outcomes.

Initiation of pyroptosis, an inflammatory form of programmed cell death, helps coordinate the release of cytokines and damage-associated molecular patterns, which tailor the adaptive immune system response to an offending agent. Pyroptosis is most often initiated through cellular inflammasomes that result in proteolytic cleavage and subsequent activation of the pore-forming protein, gasdermin D (GSDMD). Pyroptosis is not limited to GSDMD cleavage as, at a molecular level, pyroptosis is defined as a “gasdermin-induced necrotic cell death” and refers to cell death that occurs downstream of any gasdermin family member activation (1).

The gasdermin superfamily consists of six family members that arose through gene duplication events and are named gasdermins A–E and pejvakin (2, 3). With the exception of pejvakin, gasdermin proteins share a two-domain architecture consisting of a highly conserved N-terminal putative pore-forming domain and a C-terminal inhibitory domain connected by a flexible linker region (4). GSDMD is the best understood family member and is activated through cleavage by a number of proteases, including caspases-1/4/5/8, neutrophil elastase, and cathepsin G (5, 6, 7, 8, 9, 10, 11, 12). Ectopic expression of N-terminal GSDMD or cleavage of full-length GSDMD results in rapid plasma membrane permeabilization leading to ion flux and eventual lytic cell death (5, 6, 11). While GSDMD is highly present in immune cells, other members of the gasdermin family are broadly expressed and may exert tissue-specific effects (1).

Since the discovery of GSDMD as the effector of inflammasome-driven pyroptosis in 2015, studies on gasdermin family members have largely focused on the ability of these gasdermin family members to affect pyroptosis downstream of various stimuli (13, 14, 15, 16, 17, 18). While the broad mechanisms of autoinhibition and cleavage appear universal for most gasdermin family members, the lipid-binding properties, membrane and organelle preferences for pore insertion, and the kinetics of cell death relative to activation are unknown. Despite being the first identified member of the gasdermin family, GSDMA remains one of the least well-studied members. GSDMA3, a mouse homolog of GSDMA, was first identified through positional cloning of a gene causing abnormal hair and skin phenotypes in mice and named for its limited expression in the gastrointestinal tract and skin (19, 20). Later studies on GSDMA3 demonstrated that the ectopic expression of the N-terminal fragment results in mitochondrial reactive oxygen species generation, and N-terminal GSDMA is delivered to the mitochondria through interactions with HSP90 and TOM70 (21, 22). Most recently, *Streptococcal pyogenes* exotoxin B has been identified as a protease that cleaves GSDMA in keratinocytes leading to pyroptosis (17, 18). Despite the emerging studies on GSDMA, no study has reconciled the mitochondrial findings in early murine studies with the potential that GSDMA functions much like GSDMD by forming pores in the plasma membrane to cause lytic cell death.

It has been long assumed that because of their high sequence identity of the N-terminus as well as their structural similarities that the gasdermin family members all execute a lytic cell death in their respective tissues of expression (4, 5, 13). Most studies examining the pore formation of gasdermin family members in parallel have relied on transfection experiments, which may be subject to overexpression artifacts (4, 5). Given the linkage of gasdermin family members to various inflammatory diseases and widespread interest in pharmacological targeting of gasdermin family members for diverse disease settings, we sought to directly compare the well-understood family member GSDMD with the less understood family member, GSDMA (23, 24, 25, 26, 27). To this end, we developed a chimeric protein system to study the kinetic effects of GSDMA activation relative to GSDMD. We use this system to show that ectopic expression of N-terminal GSDMA and N-terminal GSDMD do not lead to equivalent cell death. We further show that after activation, GSDMD rapidly localizes to both the plasma membrane and organelles, whereas GSDMA preferentially localizes to organelles with delayed and diminished accumulation at the plasma membrane. As a consequence of the kinetics of this subcellular localization, N-terminal GSDMA results in early mitochondrial dysfunction relative to plasma membrane permeabilization. In all, this work demonstrates that not all gasdermin family members function identically, and their role in their distinct tissues of expression downstream of activation likely results in differential immunological outcomes.

## Results

### N-terminal GSDMD and N-terminal GSDMA cause variable plasma membrane permeabilization and cell lysis

**Figure 1:**
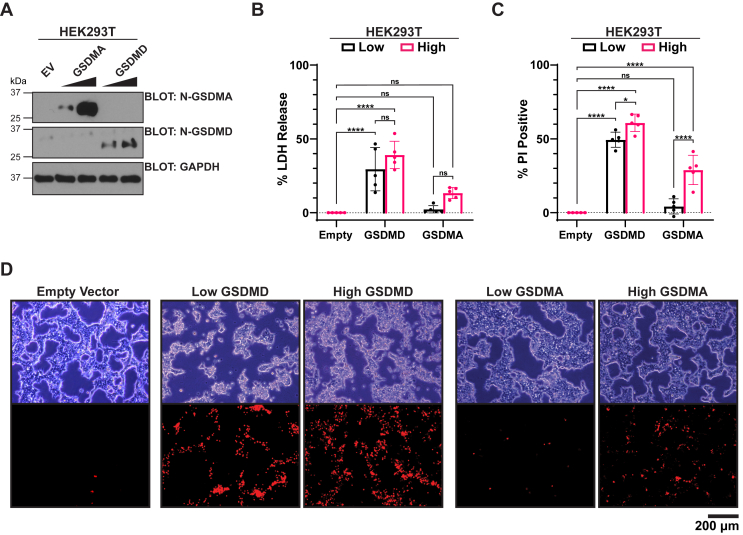
**N-terminal GSDMD and GSDMA cause variable plasma membrane permeabilization and cell lysis.***A*–*D*, HEK293T cells transfected with low concentration (1 μg) or high concentration (6 μg) N-terminal GSDMA or GSDMD. *A*, representative Western blot of transfected cells in *B*–*D*. *B*, quantification of cell lysis by lactate dehydrogenase (LDH)-release assay normalized to empty vector transfection and lysed controls. GSDMD-transfected cells show substantial cell lysis at both low and high DNA concentrations, whereas GSDMA-transfected cells show minimal cell lysis only at high concentration of DNA. *C*, quantification of gasdermin membrane permeabilization by the cell-impermeable dye propidium iodide (PI) normalized to empty vector transfection and lysed controls. GSDMD-transfected cells show substantial PI positivity at both low and high concentrations of DNA, whereas GSDMA-transfected cells show PI positivity only with high concentrations. *D*, representative bright field and fluorescent images of cells in (*B*). Graph bars represent mean ± standard deviation of biological replicates. Graph points represent pooled technical replicates per biological replicate. Statistical analyses were performed using one-way ANOVA. ∗*p* ≤ 0.05, ∗∗∗∗*p* ≤ 0.0001. Images are representative of at least five independent experiments. GSDMA, gasdermin A; GSDMD, gasdermin D; HEK293T, human embryonic kidney 293T cell line.

To directly compare the cell death–inducing properties of GSDMD and GSDMA, we created expression vectors that express N-terminal GSDMD (amino acids 1–275) or N-terminal GSDMA (amino acids 1–252). The expression vectors were transfected into human embryonic kidney 293T (HEK293T) cells at low DNA (1 μg) or high DNA (6 μg) amounts (Fig. 1*A*). Two traditional assays used to study pyroptosis, plasma membrane permeabilization measured by propidium iodide (PI) influx and cell lysis measured by lactate dehydrogenase (LDH) release into the supernatant, were then utilized. At both low and high concentrations, N-terminal GSDMD expression caused significant PI influx and LDH release. GSDMA resulted in PI influx and LDH release only at high concentrations of DNA and at lower amplitude than both low and high concentrations of GSDMD (Fig. 1, *B*–*D*). These results hint that GSDMA may not be as potent as GSDMD at effecting cell death by plasma membrane permeabilization and subsequent progression to overt cell lysis.

### A chimeric GSDMA–GSDMD protein interacts with and is cleaved by caspase-1

**Figure 2:**
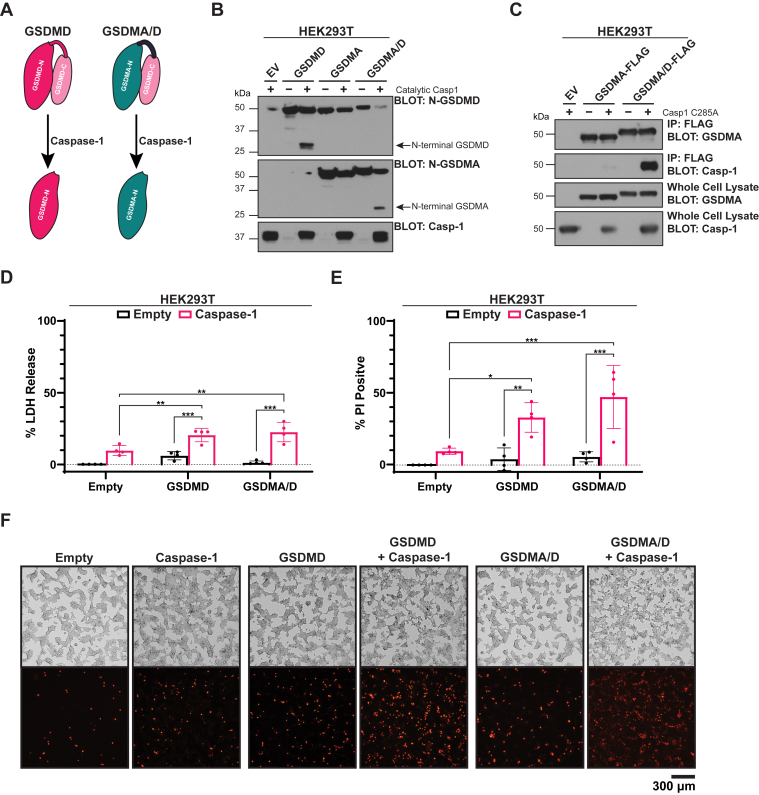
**Caspase-1 interacts with and cleaves chimeric GSDMA/D.***A*, schematic of caspase-1 cleavage of WT GSDMD and chimeric GSDMA/D. *B*, HEK293T cells transfected with empty vector, WT GSDMD, WT GSDMA, or GSDMA/D chimera with or without catalytic caspase-1. About 16 h after transfection, samples were lysed and assayed for cleavage of the full-length protein. Caspase-1 cotransfection with gasdermins results in cleavage of WT GSDMD and chimeric GSDMA/D protein but not WT GSDMA. *C*, HEK293T cells were transfected with empty vector, WT GSDMA-FLAG, or GSDMA/D-FLAG with or without catalytic dead caspase-1 (Caspase-1 C285A). About 16 h after transfection, cells were lysed, and a portion was set aside as whole-cell lysate. Remaining cell lysate was immunoprecipitated using anti-FLAG. Samples were assayed for association of caspase-1 C285A with WT GSDMA or GSDMA/D. Caspase-1 coimmunoprecipitated with the chimeric GSDMA/D but not WT GSDMA. *D*–*F*, HEK293T cells transfected with empty vector, WT GSDMD, or GSDMA/D chimera with or without catalytic caspase-1. *D*, cell lysis as measured by LDH release and normalized to empty vector transfection and lysed controls. Both GSDMD and GSDMA/D chimera result in increased LDH release when cotransfected with caspase-1 relative to caspase-1 alone. *E*, plasma membrane permeabilization as measured by PI staining and normalized to empty vector transfected and lysed controls. Both GSDMD and GSDMA/D chimera cotransfected with caspase-1 result in increased PI positivity relative to caspase-1 alone. *F*, representative fluorescent and bright field images of cells in (*E*). Graph bars represent mean ± standard deviation of biological replicates. Graph points represent pooled technical replicates per biological replicate. Statistical analyses were performed using one-way ANOVA. ∗*p* ≤ 0.05, ∗∗*p* ≤ 0.01, and ∗∗∗*p* ≤ 0.001. Images are representative of at least three independent experiments. Western blots are representative of at least three independent experiments. GSDMA, gasdermin A; GSDMD, gasdermin D; HEK293T, human embryonic kidney 293T cell line; LDH, lactate dehydrogenase; PI, propidium iodide.

While the data in Figure 1 suggest that GSDMA is not as effective as GSDMD in causing cell death, this could be due to overexpression artifacts, kinetic differences in inducing cell death, or intrinsic properties of GSDMA that lead to diminished cell death. Until recently, studies on GSDMA function have been largely limited to transient overexpression studies of the putative pore-forming domain because of lack of knowledge of an activating protease. Recent work demonstrated that *Streptococcus pyogenes* exotoxin B cleaves GSDMA in keratinocytes leading to its activation; however, an endogenous activating protease for GSDMA has yet to be identified (17, 18). Given this, we sought to develop a method by which GSDMA could be acutely activated. To this end, we leveraged our knowledge of GSDMD activation. GSDMD is well established to be activated by the inflammatory caspases, caspases 1/4/5/11, and the mechanism by which it is recognized was recently described (28, 29). The inflammatory caspases contain an exosite that recognizes residues in the C-terminus of GSDMD, leading to cleavage in the flexible linker region after aspartate-275. The C-terminus of GSDMD is both necessary and sufficient for this interaction, which allows activation of N-terminal GSDMD. We therefore created a chimeric gasdermin protein consisting of the N-terminus of GSDMA and the C-terminus of GSDMD that would allow us to activate GSDMA in an inducible and kinetic manner, hereafter referred to as GSDMA/D (Fig. 2*A*).

To test our chimeric protein system, we first cotransfected catalytic caspase-1 with wildtype GSDMD, wildtype GSDMA, or chimeric GSDMA/D protein into HEK293T cells and assayed cleavage of the full-length gasdermin proteins by Western blot. Caspase-1 cleaved GSDMD into a 30 kDa N-terminal fragment but did not cleave full-length GSDMA. We also observed significant crossreactivity between this GSDMD antibody with GSDMA. Transfection of catalytically active caspase-1 with chimeric GSDMA/D, but not transfection of GSDMA/D alone, resulted in generation of a 30 kDa GSDMA N-terminal fragment (Fig. 2*B*). To confirm this cleavage product was due to association of caspase-1 with chimeric GSDMA/D, we cotransfected catalytic dead caspase-1 with GSDMA that contained a C-terminal FLAG tag or chimeric GSDMA/D that contained a C-terminal FLAG tag. We immunoprecipitated the gasdermin proteins using FLAG and looked for coimmunoprecipitation of caspase-1. Caspase-1 effectively coimmunoprecipitated when expressed with GSDMA/D but not when expressed alone or with GSDMA (Fig. 2*C*).

We next sought to validate that cleavage of GSDMA/D produced a functional product by measurement of PI influx and LDH release. GSDMD or chimeric GSDMA/D was transfected into HEK293T cells with or without caspase-1 and then assayed for PI influx and LDH release. PI influx and LDH release occurred when wildtype GSDMD or chimeric GSDMA/D was transfected with caspase-1 but not when either construct was transfected alone (Fig. 2, *D*–*F*). No spontaneous pyroptotic activity occurred without caspase-1 activity, suggesting that both GSDMD and GSDMA/D require cleavage to be active. Unlike transfection of the p30 fragments alone where GSDMD transfection resulted in increased readouts of pyroptosis relative to GSDMA, cotransfection of caspase-1 with either GSDMD or GSDMA/D resulted in similar levels of pyroptosis. This could be due to the limitations of cotransfections including requirement of both plasmids to enter the cell as well as the kinetics of cleavage of the full-length proteins by caspase-1. These data establish that the C-terminus of GSDMD is sufficient to both autoinhibit the N-terminus of GSDMA and mediate interactions with caspase-1 leading to GSDMA/D cleavage and activation.

### Chimeric GSDMA/D is cleaved downstream of NOD-, LRR-, and pyrin domain–containing protein 3 inflammasome activation but shows delayed plasma membrane permeabilization and cell lysis

**Figure 3:**
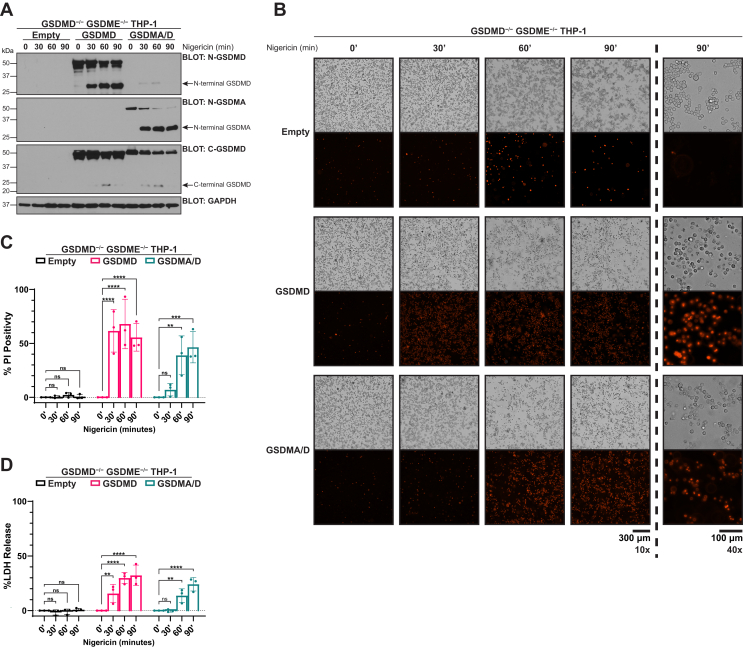
**Chimeric GSDMA/D is cleaved downstream of NLRP3 inflammasome activation but shows delayed plasma membrane permeabilization and cell lysis.***A*–*D*, GSDMD/GSDME double knockout THP-1 monocytes stably reconstituted with either empty vector, WT GSDMD, or GSDMA/D chimera and PMA differentiated for 18 h prior to experimentation. Differentiated cells were treated with 20 μM nigericin for indicated time points. GSDMD/GSDME double knockout THP-1 cells do not express any gasdermin family members. *A*, after indicated treatment times with nigericin, samples were lysed and assayed for cleavage of the full-length gasdermin proteins. GSDMD and GSDMA/D chimera showed similar cleavage kinetics with cleavage of the full-length protein by 30 min of nigericin. *B*, representative bright field and fluorescent images of THP-1 stained with propidium iodide (PI). All images taken at 10× zoom, except the rightmost panel that was taken at 40× zoom. While WT GSDMD cells showed increased PI positivity that peaked by 30 min, GSDMA/D showed delayed PI positivity that started around 60 min. *C*, after indicated treatment times with nigericin, plasma membrane permeabilization was measured by PI staining. As in *B*, WT GSDMD cells show rapid PI positivity by 30 min, whereas GSDMA/D chimeric cells show an increase in PI positivity starting at 60 min. *D*, after indicated treatment times with nigericin, cell lysis was measured by LDH release. While WT GSDMD cells showed some lysis by 30 min that peaked by 60 min, GSDMA/D chimeras showed delayed cell lysis that did not peak by the latest time point. Graph bars represent mean ± standard deviation of biological replicates. Graph points represent pooled technical replicates per biological replicate. Statistical analyses were performed using two-way ANOVA. ∗∗*p* ≤ 0.01, ∗∗∗*p* ≤ 0.001, and ∗∗∗∗*p* ≤ 0.0001. Images are representative of at least three independent experiments. Western blots are representative of at least three independent experiments. GSDMA, gasdermin A; GSDMD, gasdermin D; LDH, lactate dehydrogenase; NLRP3, NOD-, LRR-, and pyrin domain–containing protein 3; PMA, phorbol 12-myrisate 12-acetate.

To both overcome potential overexpression artifacts and kinetically study the effects of GSDMA activation relative to GSDMD, we reconstituted GSDMD/GSDME double knockout THP-1 monocytes with either wildtype GSDMD or GSDMA/D chimera. After confirming expression, cells were terminally differentiated into macrophages with phorbol 12-myrisate 12-acetate (PMA), and the NOD-, LRR-, and pyrin domain–containing protein 3 (NLRP3) inflammasome was activated with nigericin. GSDMD and GSDMA/D demonstrated similar cleavage kinetics, with cleavage noted as early as 30 min poststimulation (Fig. 3*A*). Once the kinetics of protein cleavage were established to be the same between GSDMD and GSDMA/D, we assayed traditional markers of pyroptosis. While wildtype GSDMD cleavage rapidly led to increased PI positivity that plateaued by 30 min, chimeric GSDMA/D cleavage led to relatively delayed PI positivity, which did not plateau by the latest time point (Fig. 3, *B* and *C*). Measurements of cell lysis by LDH release largely matched PI uptake, with GSDMD plateauing by 60 min and GSDMA/D showing delayed LDH release relative to GSDMD that had not plateaued by the latest time point (Fig. 3*D*). The delay in LDH release relative to PI positivity is likely because of the presence of cells with sublytic pores that allow PI influx but has not yet progressed to cell lysis as previously described for gasdermin family members (30, 31). Together, these data reveal that when compared with GSDMD, there is quantitatively more GSDMA/D cleavage relative to total GSDMA/D but decreased pyroptosis occurring downstream of cleavage.

### N-terminal GSDMA preferentially localizes to organelles rather than the plasma membrane

**Figure 4:**
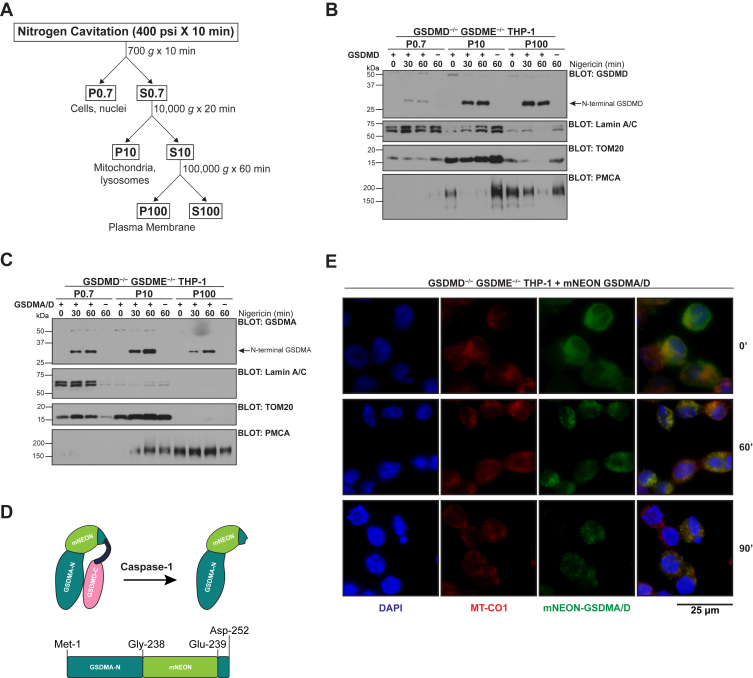
**N-terminal GSDMA preferentially localizes to organelles rather than the plasma membrane.***A*, schematic detailing nitrogen cavitation and subcellular fractionation. The P0.7 fraction represents undisrupted cells and the nuclear fraction. The P10 fraction represents the organelle fraction including mitochondria and lysosomes. The P100 fraction represents light membranes including the plasma membrane. *B*, GSDMD/GSDME double knockout THP-1 monocytes expressing no gasdermin family members were stably reconstituted with wildtype GSDMD. Cells were PMA differentiated overnight before stimulation for 0, 30, 60, or 90 min with 20 μM nigericin. After stimulus, cells were nitrogen cavitated and subcellular fractionated as described in (*A*). After cleavage, N-terminal GSDMD localizes rapidly to both the p10 and p100 fractions. *C*, GSDMD/GSDME double knockout THP-1 monocytes were reconstituted with chimeric GSDMA/D. Cells were treated as in (*B*). After cleavage, N-terminal GSDMA localizes rapidly to the organelle fraction, with diminished accumulation in the light membrane fraction, showing preference of GSDMA to accumulate in organelles rather than at the plasma membrane. *D*, design of internal mNEON-tagged GSDMA/D protein and corresponding caspase-1 cleavage product. *E*, GSDMD/GSDME double knockout THP-1 monocytes were stably reconstituted with internally tagged mNEON-GSDMA/D and PMA differentiated overnight on coverslips. After stimulation with nigericin for indicated time points, cells were fixed and stained with the mitochondrial marker MT-CO1. After counterstaining with DAPI and mounting, cells were imaged. Without stimulation, mNEON-GSDMA/D is diffusely present in the cytoplasm. After cleavage from the C terminus, mNEON-GSDMA relocalizes to a punctuated pattern that overlaps with the mitochondrial marker, MT-CO1. GSDMA, gasdermin A; GSMDD, gasdermin D; GSDME, gasdermin E; PMA, phorbol 12-myrisate 12-acetate.

A puzzling feature of the prior experiment centers on the fact that GSDMD and GSDMA/D are kinetically cleaved at identical rates, but PI uptake and LDH release are delayed and quantitatively lower with cleaved GSDMA. One explanation for this paradox could be that GSDMA targets alternative membranes, such as mitochondrial membranes, rather than the plasma membrane proper. To identify which membranes the N-terminal fragments of GSDMD and GSDMA preferentially target, THP-1 monocytes expressing either wildtype GSDMD or chimeric GSDMA/D were terminally differentiated overnight to macrophages and stimulated with nigericin. At 0, 30, 60, or 90 min poststimulation, cells were placed under high pressure nitrogen (400 psi) to disrupt the integrity of the plasma membrane but maintain the integrity of the intracellular organelles. Subsequently, cells were fractionated into a nuclear fraction, an organelle fraction, and a plasma membrane fraction (Fig. 4*A*). In concordance with early PI positivity and LDH release, N-terminal GSDMD rapidly localized to the p100 fraction that contained the plasma membrane (Fig. 4*B*). N-terminal GSDMD also rapidly localized to the p10 organelle fraction (Fig. 4*B*). Fractionation of GSDMA/D, however, revealed that N-terminal GSDMA preferentially localized in the p10 organelle fraction (Fig. 4*C*). While N-terminal GSDMA was found in the P100 fraction, it was both later and less abundant that the accumulation in the p10 fraction suggesting that N-terminal GSDMA preferentially localizes to organelles rather than the plasma membrane, whereas GSDMD localizes to both organelles and the plasma membrane equally. In all fractions, there was an enrichment of the N-terminal P30 fragment with little full-length protein, consistent with previous work showing that with the exception of GSDMB, full-length gasdermin family members do not associate with phospholipids (4, 32). This subcellular localization of N-terminal GSDMA provides a possible explanation for diminished PI positivity and LDH release relative to GSDMD and suggests that GSDMA may effect cellular dysfunction and death alternatively than direct cell lysis.

Following the subcellular fractionation experiments, we sought to determine which organelle GSDMA was preferentially targeting. Previous work has shown that GSDMD and GSDME target the mitochondria as well as murine GSDMA3 (21, 33, 34). We therefore hypothesized that this is organelle GSDMA could be preferentially targeting. To follow GSDMA localization after cleavage from the inhibitory C-terminal domain, we inserted an mNEON fluorescent tag (Fig. 4*D*). Tagging GSDMD N-terminally results in inhibition of function, and we therefore inserted the fluorescent tag into the flexible linker region of our chimeric GSDMA/D protein in an area previously determined to not interfere with function of GSDMD to preserve function of GSDMA (4, 11, 12, 35). After stable expression was verified, mNEON-GSDMA/D-expressing cells were stimulated with nigericin before fixation and staining with an anti-MT-CO1 antibody to mark the mitochondria. At baseline, mNEON-GSDMA was present diffusely in the cytoplasm. By 60 min, mNEON-GSDMA was present in a punctuated pattern that overlapped significantly with the mitochondria (Fig. 4*E*). This experiment gives further evidence that our subcellular fractionation data that GSDMA is targeting organelles, specifically the mitochondria.

### N-terminal GSDMA expression results in early mitochondrial dysfunction

**Figure 5:**
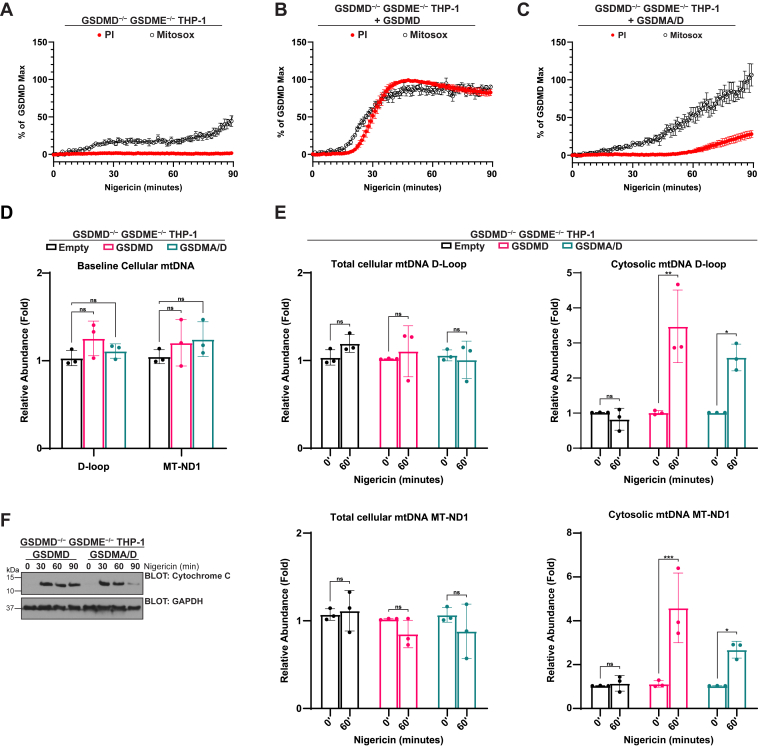
**N-terminal GSDMA expression results in early mitochondrial dysfunction.***A*–*F*, GSDMD/GSDME double knockout THP-1 monocytes were reconstituted with empty vector control, WT GSDMD, or chimeric GSDMA/D as indicated. Cells were PMA differentiated overnight before treatment. *A*, gasdermin knockout cells were stimulated with 20 μM nigericin, and PI influx (*red*) or mitochondrial superoxide generation (*black*) was measured kinetically. *B*, cells reconstituted with WT GSDMD were stimulated with 20 μM nigericin, and PI influx (*red*) or mitochondrial superoxide generation (*black*) was measured kinetically. PI positivity and Mitosox positivity occurred at identical rates, showing N-terminal GSDMD disruption of both the plasma and mitochondrial membranes. Data points were normalized to untreated cells and GSDMD maximum fluorescent readouts. *C*, cells reconstituted with the GSDMA/D chimera were stimulated with 20 μM nigericin, and PI influx (*red*) or mitochondrial superoxide generation (*black*) was measured kinetically. Relative to PI influx, there was increased Mitosox fluorescence at earlier time points indicating mitochondrial membrane disruption prior to plasma membrane disruption. Data points were normalized to untreated cells and GSDMD maximum fluorescent readouts. *D*, baseline total cellular mitochondrial DNA of empty vector, GSDMD reconstituted, and GSDMA/D reconstituted cells relative to empty vector. *E*, after stimulation with 20 μM nigericin, total cellular mitochondrial DNA (*left*) and mitochondrial DNA release into the cytoplasm of the cell (*right*) were measured relative time 0 per each genotype. By 60 min, both GSDMD and GSDMA/D cells showed significant increase in mitochondrial DNA present in the cytoplasm relative to empty vector control cells. *F*, representative Western blot of cytosolic fraction. After indicated treatment times, GSDMA/D chimera and WT GSDMD cellular lysates were fractionated and assayed for cytochrome *c* release into the cytosol from the mitochondria. Both GSDMD and GSDMA result in cytochrome *c* release into the cytosol. Graph bars represent mean ± standard deviation of at least three biological replicates. Graph points represent pooled technical replicates per biological replicate. Statistical analyses were performed using two-way ANOVA. ∗*p* ≤ 0.05, ∗∗*p* ≤ 0.01, and ∗∗∗*p* ≤ 0.001. GSDMA, gasdermin A; GSDMD, gasdermin D; GSDME, gasdermin E; PI, propidium iodide; PMA, phorbol 12-myrisate 12-acetate.

If cleaved GSDMA is preferentially targeting mitochondrial membranes rather than the plasma membrane, then cells with active GSDMA should exhibit signs of mitochondrial dysfunction earlier than plasma membrane disruption. Unlike GSDMD, which targets all cellular membranes and therefore shows equivalent kinetics of plasma membrane disruption and mitochondrial dysfunction (Fig. 5*B*), GSDMA shows faster mitochondrial superoxide generation relative to PI uptake (Fig. 5*C*). Following this, we examined if gasdermin disruption of the mitochondria resulted in mitochondrial DNA (mtDNA) release into the cytosol. First, we confirmed that our empty vector, GSDMD, and GSDMA/D reconstituted cells contained similar levels of mtDNA at baseline (Fig. 5*D*). Next, cells were stimulated with nigericin, and mtDNA in the cytosol was measured relative to baseline. Cleaved GSDMD and GSDMA both resulted in increased mitochondrial DNA in the cytosol, whereas empty vector cells did not, indicating that disruption of the mitochondria by both gasdermins is sufficient to release mtDNA (Fig. 5*E*). Finally, we assayed cytochrome *c* release from the mitochondria and saw that by 30 min, there was cytochrome *c* release from both GSDMD and chimeric GSDMA/D cells (Fig. 5*F*).

### Apoptotic consequences of mitochondrial dysfunction are not necessary for progression to cell death in GSDMA-mediated pyroptosis

**Figure 6:**
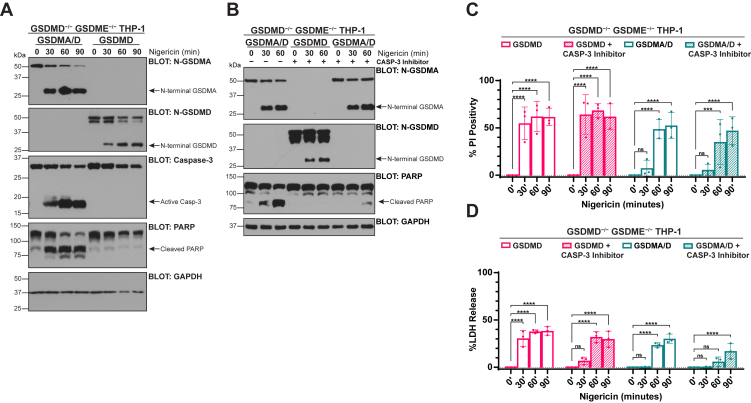
**Apoptotic consequences of mitochondrial dysfunction are not necessary for progression to cell death in GSDMA-mediated pyroptosis**. *A*–*D*, GSDMD/GSDME double knockout THP-1 monocytes were reconstituted with empty vector control, WT GSDMD, or chimeric GSDMA/D as indicated. Cells were PMA differentiated overnight before treatment. *A*, after indicated treatment times, GSDMA/D chimera and WT GSDMD cellular lysates were assayed by Western blot for caspase-3 and PARP cleavage. GSDMD reconstituted cells do not transition to apoptosis, whereas GSDMA/D cells show a transition to apoptosis as indicated by caspase-3 and PARP cleavage. *B*, GSDMD or GSDMA/D reconstituted cells were treated with Z-DEVD-FMK, a caspase-3 inhibitor. Treatment with Z-DEVD-FMK results in caspase-3 inhibition as indicated by lack of PARP cleavage in these cells. *C*, GSDMD or GSDMA/D reconstituted cells were treated with or without Z-DEVD-FMK, and PI positivity was measured at indicated time points. Inhibition of caspase-3 does not inhibit plasma membrane permeabilization by GSDMD or GSDMA. *D*, GSDMD or GSDMA/D reconstituted cells were treated as in (*D*), and LDH release was measured at indicated time points. Inhibition of caspase-3 does not inhibit lytic cell death mediated by GSDMD or GSDMA. Graph bars represent mean ± standard deviation of biological replicates. Graph points represent pooled technical replicates per biological replicate. Statistical analyses were performed using two-way ANOVA. ∗*p* ≤ 0.05, ∗∗*p* ≤ 0.01, ∗∗∗*p* ≤ 0.001, and ∗∗∗∗*p* ≤ 0.0001. Images are representative of at least three independent experiments. Western blots are representative of at least three independent experiments. GSDMD, gasdermin D; GSDME, gasdermin E; LDH, lactate dehydrogenase; PARP, poly(ADP-ribose) polymerase; PI, propidium iodide; PMA, phorbol 12-myrisate 12-acetate.

Because of early mitochondrial disruption relative to plasma membrane disruption and cytochrome *c* release, we hypothesized that GSDMA was perhaps initiating cell death through an apoptotic pathway. To formally test this, we assayed caspase-3 and poly(ADP-ribose) polymerase (PARP) cleavage downstream of GSDMD or chimeric GSDMA/D cleavage. Despite cytochrome *c* release into the cytosol occurring in both genotypes, no markers of apoptosis were noted when GSDMD was activated (Fig. 6*A*). In the chimeric GSDMA/D cells however, a shift to an apoptotic state indicated by caspase-3 cleavage and PARP cleavage was observed (Fig. 6*A*). Since cytochrome *c* release occurred in both cases, we hypothesized that the delayed formation of plasma membrane pores in the GSDMA/D-expressing cells allowed the release of cytochrome *c* from the mitochondria to facilitate the formation of the apotosome, whereas GSDMD-expressing cells progressed to lytic cell death before this occurred.

To determine if this shift to apoptosis is responsible for cell death in GSDMA-mediated pyroptosis, we used a caspase-3 inhibitor, Z-DEVD-FMK, to inhibit apoptosis from occurring, while still allowing caspase-1 to be active to cleave our chimeric protein (Fig. 6*B*). As expected, other than a slight delay most likely because of partial caspase-1 inhibition, PI uptake and LDH release were not altered in GSDMD-expressing cells treated with Z-DEVD-FMK, as apoptosis is not initiated during GSDMD-mediated pyroptosis (Fig. 6, *C* and *D*). Similar to GSDMD, GSDMA/D-expressing cells still displayed PI positivity and LDH release even when caspase-3 is inhibited (Fig. 6, *D* and *E*). These data suggest that although active GSDMA leads to earlier mitochondrial dysfunction, later stage localization to the plasma membrane (Fig. 4*C*) is sufficient to cause cellular lysis, and activation of the apoptotic pathway is not necessary for continuation to lytic cellular demise.

## Discussion

Since the identification of GSDMD as the canonical executer of inflammasome-initiated pyroptosis, there has been a general assumption that all gasdermin family members function similarly to GSDMD downstream of their activating stimuli in the tissues that they are expressed. In this article, we show that equivalent expression of GSDMA and GSDMD do not result in equivalent lytic cell death. We then use a novel chimeric protein system to kinetically study the outcome of gasdermin family member activation and dissect the immediate consequences of GSDMA and GSDMD activation. While GSDMD rapidly localizes to both the plasma membrane and organelles, GSDMA preferentially localizes to organelles, with delayed and diminished localization to the plasma membrane. This organelle localization manifests as early mitochondrial dysfunction relative to plasma membrane disruption, resulting in reactive oxygen species generation, mtDNA release into the cytosol, and a transition to apoptosis prior to lytic cell death.

The mechanism of preferential targeting of GSDMA to the mitochondrial membrane over the plasma membrane remains a question and could be due to different lipid-binding properties of GSDMD and GSDMA. While previous studies on the lipid-binding properties of GSDMA and GSDMD have revealed that the most common lipids bound are cardiolipin and phosphatidylinositol phosphates, N-terminal GSDMA showed weaker binding to phosphoinositides than N-terminal GSDMD and strong binding to cardiolipin (4, 11). These lipid-binding preferences could reflect the subcellular localization preferences of the proteins, as phosphoinositides are found highly concentrated in the plasma membrane, whereas cardiolipin is found exclusively in the mitochondria. Cardiolipin is synthesized in the mitochondria and found in highest percentages in the inner mitochondrial membrane and is redistributed to the outer mitochondrial membrane (OMM) during mild mitochondrial damage (36, 37). Whether the small percentage of cardiolipin on the OMM is sufficient for GSDMA binding or if GSDMA is delivered into the mitochondria by chaperone proteins to form pores has not been determined. Studies on the murine ortholog of GSDMA, GSDMA3, demonstrated that GSDMA3 is delivered to the mitochondria through interactions with HSP90 and TOM70, and similar chaperone mechanisms should be investigated in subsequent studies for GSDMA (21). After initial mitochondrial damage, it is possible that the cardiolipin membrane rearrangement to the OMM creates a feedforward loop that increases GSDMA-mediated damage. GSDMD activation and signaling has been previously shown to engage in crosstalk with another gasdermin family member, GSDME (30, 38). GSDMA targeting of the mitochondria may result in another pathway to gasdermin family member crosstalk as caspase-3 activation will cleave GSDME (16, 30, 34) and oxidized mtDNA has been shown to activate the NLRP3 inflammasome during apoptosis (39, 40). Endogenous GSDMA activation that is at insufficient levels to cause lytic cell death but sufficient to cause mitochondrial dysfunction may result in cell lysis *via* activation of GSDME or through activation of NLRP3 and canonical GSDMD-mediated pyroptosis.

Our data indicate that GSDMA preferentially targets the mitochondria with the consequence of activating apoptosis but ultimately results in lytic cell death by accumulation at the plasma membrane (Fig. 6). One hypothesis for why this occurs is that although in our system GSDMA expression levels are high enough to cause lytic cell death, this may not be the case for all cell types. Our laboratory and others have shown that low expression of gasdermin family members is not sufficient to cause cell death, and extensive membrane repair occurs as the cell attempts to repair membrane pores (30, 31, 41). Activating apoptosis in addition to direct GSDMA-mediated cell death could be a mechanism to ensure the cell ultimately dies. Another hypothesis is that early GSDMA accumulation at the mitochondria results in accumulation of mtDNA in the cytosol, such that upon plasma membrane rupture, substantial quantities of mtDNA are released into the extracellular space. mtDNA is a well-recognized damage-associated molecular pattern that participates in the propagation of the inflammatory response in other cell types and pathogenesis of inflammatory disease such as scleroderma (systemic sclerosis [SSc]) (42, 43, 44, 45). As GSDMA has been identified as a susceptibility gene to SSc with increased expression seen in patients with SSc, the link between GSDMA and extracellular mtDNA in these patients should be investigated (46, 47).

In conclusion, we have created a novel system to study gasdermin family members in an inducible and kinetic manner and revealed that unlike GSDMD that executes a rapid lytic cell death, GSDMA preferentially targets the mitochondrial membrane resulting in mitochondrial dysfunction, reactive oxygen species generation, and mtDNA release. Our findings help better understand gasdermin family biology, as GSDMD has been used as a model for all gasdermin family members. The intracellular targeting of mitochondria prior to delayed lytic cell death may lead to increased inflammatory signaling, resulting in an amplified immune response during GSDMA activation.

## Experimental procedures

### Cell lines and plasmids

HEK293T used for transient transfections and virus production were purchased from American Type Culture Collection and cultured in Dulbecco's modified Eagle's medium (Corning) supplemented with 10% supercalf serum (Gemini) and 1% antibiotics/1% antimycotics (100× anti-anti; Gibco). THP-1 monocytes were purchased from American Type Culture Collection and cultured in RPMI (Corning) supplemented with 10% fetal bovine serum (Gemini) and 1% anti-anti. All cells were routinely tested for mycoplasma contamination by PCR.

Plasmids for transient transfection were Gibson subcloned into an in-house-generated plasmid. Plasmids for reconstituted THP-1 monocytes were Gibson subcloned into a lentiviral expression plasmid as previously described (48). pMD2.6 (Addgene plasmid 12259) and PsPax2 (Addgene plasmid 12260) were a gift from Didier Trono.

Generation of *GSDMD*^*−/−*^ *GSDME*^*−/−*^ THP-1 monocytes was previously described (30). GSDMD, GDSMA, and GSDMA/D chimera reconstituted THP-1 monocytes were made using lentiviral transduction. Lentivirus was produced in HEK293T cells using calcium phosphate transfection of desired plasmid cotransfected with PsPAX and PMD.2 at a ratio of 4:3:1. Two days after transfection, supernatant was harvested, centrifuged at 350*g* for 5 min, and filtered through a 0.45 μm filter and then added directly to *GSDMD*^*−/−*^ *GSDME*^*−/−*^ THP-1 monocytes with 10 μg/ml Polybrene transfection reagent (MilliporeSigma). Two days after addition of lentivirus, cells were selected in 1 mg/ml G418 (InvivoGen). Reconstitution of cells was verified by Western blot.

### Transient transfections

For transient transfection experiments, HEK293T cells were plated in 6-well dishes and transfected using calcium phosphate. DNA concentrations were consistent across cell lines by use of an empty-vector promoter control. About 4 h after transfection, cells were washed 1× with PBS, and fresh media were added. Cells were incubated for 18 h and collected the next day for analysis.

### Inflammasome activation

THP-1 cells were differentiated into adherent macrophages using 100 ng/ml PMA (MilliporeSigma) for 18 to 20 h. NLRP3 inflammasome was activated with 20 μM nigericin (MilliporeSigma). Inflammasome activation was conducted in Live Cell Imaging Solution (LCIS) (Thermo Fisher Scientific) in 0.5 ml per 24-well dish, 1.5 ml per 60 mm plate, or 5 ml per 10 mm plate. For caspase-3 inhibition studies, cells were incubated in 10 μM Z-DEVD-FMK (Selleckchem; catalog no.: S7312) for 30 min prior to inflammasome stimulation.

### PI influx, LDH release, and mitosox assays

For PI assays of transient transfections, 1 μg/ml PI (Molecular Probes) was added to the cells 30 min prior to imaging and analysis. PI fluorescence (excitation/emission, 533/617) was measured in each well on Spectramax i3x Multi Mode microplate reader (Molecular Devices). Relative PI uptake was calculated for each well as (fluorescence of well – fluorescence of untransfected cells)/(fluorescence of lysed well – fluorescence of untransfected cells). For THP-1 experiments, cells were differentiated as described previously. Prior to start of assay, cells were washed once with PBS, and LCIS supplemented with 1 μg/ml PI was added to each well. For kinetic PI assays, fluorescence of every well was measured kinetically at 1 min intervals on Spectramax i3x Multimode microplate reader maintained at 37°. Maximum fluorescence was obtained by lysing each well with 0.1% Triton X-100, and relative PI uptake was calculated as described previously.

Cytotoxicity assays were measured by LDH release. For transient transfections, LDH release was measured 18 h after transfection. For all other assays, 0.5 × 10^6^ THP-1 monocytes were PMA differentiated overnight. Cells were washed 1× with PBS, and media were replaced with LCIS as aforementioned. After nigericin stimulation, LDH release was measured at indicated times. Background and maximum LDH was measured from untreated and lysed cells, respectively. LDH release was measured using CyQUANT LDH Cytotoxicity Assay (Thermo Fisher Scientific) according to the manufacturer’s instructions.

For measurement of superoxide species, THP-1 monocytes were differentiated as described previously, and media were replaced with LCIS supplemented with 5 μM mitosox (Thermo Fisher Scientific). Fluorescence was measured kinetically at 1 min intervals (excitation/emission, 510/580 nm) on a Spectramax i3x Multi Mode microplate reader held at 37°. Relative fluorescence was calculated by subtracting untreated well at each time point for each genotype.

### Cell imaging

For Figure 1, brightfield and epifluorescent imaging was conducted using a 10× objective magnification on a DM Il LED microscope (Leica). For all other figures, brightfield and fluorescent imaging was conducted using a Cytation 5 Imaging Reader (Biotek).

### Nitrogen cavitation and subcellular fractionation

Differentiated THP-1 cells were treated with nigericin for indicated time points. Cells were scraped in supernatant and resuspended in ice-cold fractionation buffer (250 mM sucrose, 20 mM Hepes [pH 7.4], 10 mM KCl, 1.5 mM MgCl_2_, 1 mM EDTA, 1 mM EGTA, protease inhibitor cocktail, and PMSF. Cells were then subjected to nitrogen cavitation to maintain integrity of intracellular granules at 400 psi for 10 min. The cavitate was then released to disrupt plasma membranes and centrifuged at 700*g*, 10,000*g*, and 100,000*g* to obtain subcellular fractions representing the nuclear, organelle, and plasma membrane fractions, respectively. Fractions were then run on Western blot as described later.

### Immunofluorescence

Glass coverslips in 24-well plates were coated with 200 μl of 0.01% poly-l-ornithine solution. After aspiration and drying, 0.5 × 10^6^ cells in 500 μl of media were plated and differentiated with PMA for 18 h. Cells were washed 1× with PBS, and media were replaced with LCIS. After 20 μM nigericin stimulation for indicated time points, 500 μl 4% formaldehyde in PBS was added directly to culture medium for 2 min. After 2 min, the prefixation culture medium was replaced with 500 μl of 2% formaldehyde in PBS for 20 min. Cover slips were washed 3× with PBS and then blocked and permeabilized for 1 h in PBS containing 1% bovine serum albumin (BSA), 3% goat serum, and 0.3% Triton X-100. Coverslips were then incubated overnight at 4° overnight with primary antibody in PBS containing 1% BSA and 0.3% Triton X-100. Primary antibodies used were anti-MT-CO1, 1:1000 dilution (Invitrogen; catalog no.: 459600). Primary antibodies were visualized by staining with Alexa Fluor 594–conjugated goat antimouse immunoglobulin G (Invitrogen; catalog no.: A11005). Coverslips were counterstained and mounted with ProLong Diamond Antifade Mountant with 4′,6-diamidino-2-phenylindole (Invitrogen; catalog no.: P36962) and imaged using an Olympus IX73 inverted microscope system with 4′,6-diamidino-2-phenylindole, FITC, and tetramethylrhodamine filters at 60× objective.

### Cytochrome *c* and mtDNA release

Measurement of cytochrome *c* and mtDNA release into the cytosol was adapted from Bryant *et al.* (49). THP-1 monocytes were differentiated overnight and stimulated with nigericin as described previously. After indicated times, cells were scraped directly in supernatant and spun at 200*g* for 5 min. Cell pellets were washed 1× with Dulbecco’s PBS and divided into two tubes. One tube was lysed as whole-cell extract in SDS lysis buffer (20 mM Tris, pH 8, 1% [v/v] SDS, and protease inhibitors). The other tube was lysed in digitonin lysis buffer (50 mM Hepes, pH 7.4, 150 mM NaCl, 10 μg/ml digitonin, and protease inhibitors) and incubated for 10 min before centrifugation at 950*g* for 5 min. Supernatant was taken as the cytosolic fraction, and pellet was lysed in NP-40 lysis buffer (50 mM Tris, pH 7.5, 150 mM NaCl, 1 mM EDTA, 1% [v/v] NP-40, 10% [v/v] glycerol, and protease inhibitors). After lysis, pellet was centrifuged at maximum speed for 10 min, and the supernatant was kept as mitochondrial extract. The remaining cell pellet was lysed in SDS lysis buffer and was representative of the nuclear extract. From each fraction, a portion was kept for Western blot, and the rest was used for phenol–chloroform DNA extraction. Following DNA extraction, DNA in fractions was detected by quantitative PCR. mtDNA abundance relative to nuclear DNA was first calculated for whole-cell extracts using the ΔΔCq method and expression normalized to empty vector. Following this, cytosolic mtDNA abundance was calculated as follows: ΔCq = (Cq D-loop cytosol) − Cq (ΔCq WCE). ΔΔCq = ΔCq − median ΔCq of time 0. Relative abundance of cytosolic mtDNA = 2^−(ΔΔCq)^. The primer sequences are as follows: KCNJ10, forward: 5′-GCGCAAAAGCCTCCTCATT-3′ and reverse: 5′-CCTTCCTTGGTTTGGTGGG-3′; MT-ND1, forward: 5′-GAACTAGTCTCAGGCTTCAACATCG-3′ and reverse: 5′-CTAGGAAGATTGTAGTGGTGGAGGGTG; MT-D-loop, forward: 5′-CATAAAGCCTAAATAGCCACACG-3′ and reverse 5′-CCGTGAGTGGTTAATAGGGTGATA-3′.

### Immunoprecipitations and Western blots

For immunoprecipitation experiments, cells were scraped with supernatant and centrifuged at 17,000*g* for 1 min. Cells were washed with PBS and lysed with Triton lysis buffer (150 mM NaCl, 50 mM Tris [pH 7.4], 2.5 mM sodium pyrophosphate, 1 mM β-glycerophosphate, 5 mM iodoacetamide, 5 mM *N*-ethylmaleimide, 1 mM PMSF, 1 mM sodium orthovanadate, 1 mM EGTA, 1 mM EDTA, 1% Triton X-100, and complete protease inhibitor cocktail). After lysis, lysates were centrifuged at 17,000*g*, and cellular debris was discarded. Immunoprecipitation was performed by incubating lysates with Anti-FLAG M2 Affinity gel (MilliporeSigma) overnight. Immunoprecipitants were washed three times in lysis buffer before boiling for 5 min at 95 °C in lysis buffer and Laemmli sample buffer diluted to 1×.

For Western blots, cells were scraped with supernatant from plates and centrifuged at 17,000*g* for 1 min. Cell pellets were washed with PBS and lysed with SDS lysis buffer (1% SDS, 1 mM EDTA, 0.01 M Tris [pH 8], and 150 mM NaCl). Laemmli buffer containing 1% final beta-mercaptoethanol was added to 1×, and samples were boiled for 5 min at 95 °C.

Lysates were separated using SDS-PAGE on 8 to 15% acrylamide gels depending on protein size. Proteins were transferred onto 0.22 μm nitrocellulose membranes. Membranes were washed with Tris-buffered saline with Tween-20 (TBST) and blocked in 5% nonfat dry milk diluted in TBST for 1 h. Membranes were then incubated in primary antibody diluted 1:1000 in either 5% nonfat dry milk or 5% BSA diluted in TBST overnight at 4 °C. Membranes were washed three times in TBST and incubated with horseradish peroxidase–conjugated secondary antibody diluted 1:10,000 in 5% nonfat dry milk diluted in TBST for 2 h. Membranes were washed in TBST and developed with enhanced chemiluminescence.

All primary antibodies were used at 1:1000 dilution. Antibodies used were Caspase-1 (catalog no.: ab179515; Abcam), GSDMD (catalog no.: HPA044487; Atlas Antibodies; of note, this exhibited significant crossreactivity with GSDMA), GSDMD (catalog no.: 97558; Cell Signaling Technology), GSDMD C-terminal (catalog no.: ab228824; Abcam), GSDMA (catalog no.: ab2307678; Abcam), GAPDH (catalog no.: 60004-1-Ig; Proteintech), TOM20 (catalog no.: CST42406; Cell Signaling Technology), Caspase-3 (catalog no.: 9662s; Cell Signaling Technology), Lamin A/C (catalog no.: ab238303; Abcam), PMCA (catalog no.: ab190355; Abcam), and PARP (catalog no.: 9542; Cell signaling Technology).

## Data availability

All data are contained within the article.

## Conflict of interest

The authors declare that they have no conflicts of interest with the contents of the article.
